# Trauma induced coagulopathy is limited to only one out of four shock induced endotheliopathy (SHINE) phenotypes among moderate-severely injured trauma patients: an exploratory analysis

**DOI:** 10.1186/s13049-024-01236-8

**Published:** 2024-08-19

**Authors:** Pär I. Johansson, Martin Vigstedt, Nicola S. Curry, Ross Davenport, Nicole P. Juffermans, Simon J. Stanworth, Marc Maegele, Christine Gaarder, Karim Brohi, Jakob Stensballe, Hanne H. Henriksen, for the Targeted Action for Curing Trauma-Induced Coagulopathy (TACTIC) Collaborators

**Affiliations:** 1 CAG Center for Endotheliomics, Copenhagen University Hospital – Rigshospitalet, Blegdamsvej 9, 2100 Copenhagen, Denmark; 2 Department of Clinical Immunology, Copenhagen University Hospital – Rigshospitalet, Copenhagen, Denmark; 3 Department of Clinical Medicine, University of Copenhagen, Copenhagen, Denmark; 4 Oxford Haemophilia and Thrombosis Centre, Oxford University Hospitals NHS Foundation Trust, Oxford, UK; 5 Radcliffe Department of Medicine, Oxford University, Oxford, UK; 6 Trauma Sciences, Blizard Institute, Barts and the London School of Medicine and Dentistry, Queen Mary University of London, London, UK; 7 Department of Intensive Care Medicine, Amsterdam UMC, University of Amsterdam, Amsterdam, The Netherlands; 8 NHS Blood and Transplant, John Radcliffe Hospital, Oxford University Hospitals NHS Foundation Trust, Oxford, UK; 9 Department of Traumatology and Orthopaedic Surgery, Cologne-Merheim Medical Center, University of Witten/Herdecke, Cologne, Germany; 10 Department of Traumatology, Oslo University Hospital, Oslo, Norway; 11 Department of Anesthesiology and Trauma Center, Copenhagen University Hospital – Rigshospitalet, Copenhagen, Denmark

**Keywords:** Trauma, Shock induced endotheliopathy, Hierarchical clustering, Coagulopathy

## Abstract

**Background:**

Trauma induced coagulopathy remains to be an important cause of high transfusion requirements and mortality and shock induced endotheliopathy (SHINE) has been implicated.

**Methods:**

European multicenter observational study of adult trauma patients with injury severity score ≥ 16 arriving within 2 h from injury to the trauma centers. Admission blood samples obtained were used for analysis of the SHINE biomarkers (syndecan-1, soluble thrombomodulin, adrenaline) and extensive analysis of coagulation, -and fibrinolytic factors together with collection of clinical data. Hierarchical clustering of the SHINE biomarkers was used to identify the SHINE phenotypes.

**Results:**

The 313 patients clustered into four SHINE phenotypes. Phenotype 2, having the highest glycocalyx shedding, encompassing 22% of the whole cohort, had severe coagulopathy with lower levels of prothrombin, FV, IX, X, XI and severe hyperfibrinolysis with higher plasmin – alpha 2-antiplasmin (PAP) – and tPA levels and lower alpha2 – antiplasmin levels. This phenotype had significantly higher transfusion requirements and higher mortality (39% vs. 23%, 15% and 14%) but similar injury severity score (ISS) compared to the others phenotypes.

**Conclusions:**

Hierarchical clustering identified four SHINE phenotype in a cohort of trauma patients. Trauma induced coagulopathy was confined to only one of the SHINE phenotypes, encompassing 22% of the total cohort. This phenotype was characterized by severe hypocoagulability and hyperfibrinolysis, which translated to significantly higher transfusion requirements and higher mortality compared to the other SHINE phenotypes with similar injury severity, warranting further investigation.

## Background

Major hemorrhage after trauma is estimated to be responsible for nearly half of the annual 4.6 million injury deaths worldwide [1]. Up to 50% of critically bleeding patients will die, either early from exsanguination or later from multiple organ dysfunction or associated traumatic brain injury (TBI) [2–5]. Coagulopathy in trauma patients, as evaluated by activated partial thromboplastin time (APTT), prothrombin time (PT), or viscoelastic whole blood tests such as rotational thrombelastometry (ROTEM®) has consistently been reported to be associated with increased transfusion requirements and high mortality [6]. Shock-induced endotheliopathy (SHINE) has also been introduced as a contributor to the high mortality in critically ill patients, including trauma, secondary to its contribution to both coagulopathy and development of multiorgan failure [7, 8]. The SHINE pathophysiology involves the sympathetic nervous system with release of high levels of catecholamines and development of three main types of endotheliopathies: Damage/loss of the endothelial glycocalyx, cleavage of soluble thrombomodulin (sTM) with impairment of the natural protein C anticoagulant system and ultimately increased permeability due to loss of the integrity of endothelial intercellular junctions [7].

In trauma patients damage/shedding of the endothelial glycocalyx, a hydrated gel-like structure that coats the luminal surface of the endothelial cells (EC) has been reported [9–11]. Elevated levels of syndecan-1, as a measure of glycocalyx shedding, was reported to be an independent predictor of 30-day mortality, and despite comparable injury severity among trauma patients, those with high syndecan-1 levels had threefold higher mortality pointing towards a potential inherent genetic contribution. Also, high glycocalyx shedding in trauma patients has consistently has been associated with increased transfusion requirements [9–11] and apart from endogenous heparinization, secondary to the release of heparin sulphate from the damaged glycocalyx, no mechanistic explanation for the hypocoagulability observed by TEG and aPTT/PT has been provided [12]. Several of the reports published concerning syndecan-1 levels and, thereby, the glycocalyx shedding in trauma also find that sTM is significantly increased in non-survivors compared to survivors suggesting that impairment of the protein C system also may be involved in the pathophysiology [9–13].

The reports referred to above comes from linear statistic models, mainly on dichotomized data sets, which provides average effects and, potentially, prevents the identification of sub-groups of patients with differential responses [9–14]. We hypothesize that a data-driven non-linear statistical approach using unsupervised bioinformatic methods applied on the three different components involved in SHINE, i.e. adrenaline, syndecan-1 and sTM, may provide novel insight to the observed differences in coagulopathy, transfusion requirements and mortality associated with high glycocalyx shedding among severely injured trauma patients [15].

## Methods

### Study design

A retrospective multicenter observational cohort study, the Activation of Coagulation and Inflammation in Trauma-2 (ACIT-2) study [6]. Patients were excluded from the study if they arrived > 2 h post-injury; were transferred from another hospital; received more than 2000 ml crystalloid pre-hospital; or had sustained burns of over 5% of their body surface area. Pre-hospital tranexamic acid and/or blood product transfusion prior to hospital arrival was allowed. Patients were retrospectively excluded if they declined to give consent to the use of their research samples, had severe liver disease, a known pre-existing bleeding diathesis, or were taking anticoagulant medication (excluding aspirin) pre-injury.

### Patient characteristics

313 out of 2019 patients were included from the ACIT study recruited between January 2008 and July 2014, all adults (≥ 18 years old) and moderately to severely injured (Injury Severity Score (ISS) ≥ 16), who had endothelial biomarker data (syndecan-1, soluble thrombomodulin, and adrenaline) and measurements of 13 selected single nucleotide polymorphisms (SNP) of the β2-adrenergic receptor, thrombomodulin, endothelial protein C receptor (EPCR), heparanase, E-selectin and Fibrinogen. The availability of SNP´s determined the number of patients included.

Research personnel at each center screened and enrolled patients. Data were collected prospectively and included patient demographics, time of injury, mechanism of injury (blunt or penetrating), TBI (AIS Head > 3), ISS, vital signs on-scene and on arrival in the emergency department, total number of blood products, and volume of intravenous fluids administered within the first 12 h from injury. Patients were observed for 28 days from injury for the occurrence of venous thromboembolic events (deep vein thrombosis or pulmonary embolism), and overall mortality.

### Blood sampling

The blood samples were drawn within 20 min of the patient’s arrival in the emergency department including prothrombin time (PT), INR, arterial blood gas analysis.

For thromboelastometry (ROTEM®) analysis, a 2.7 ml citrated vacutainer (0.109 Molar/3.2% sodium citrate; Becton, Dickinson and Company, Plymouth, UK) was collected. Blood for coagulation and fibrinolysis protein assays was collected in a 4.5 ml glass citrated vacutainer (0.109 Molar/3.2% sodium citrate; Becton, Dickinson and Company, Plymouth, UK). The filled 4.5 ml vacutainer was centrifuged within 1 h of collection, and double-spun plasma subsequently stored at − 80 °C.

### Viscoelastic hemostatic analyses

Functional hemostatic analysis was performed within one hour of blood draw at 37 °C on a ROTEM® delta instrument (Tem International GmbH, Munich, Germany) using the automated electronic pipette according to the manufacturer’s instructions. The methodology and parameters of ROTEM® have been described previously [16].

### Biomarkers of SHINE, coagulation, and fibrinolysis

Plasma stored at − 80 °C was thawed to 37 °C immediately before all analyses. The soluble biomarkers adrenaline, syndecan-1, soluble thrombomodulin, E-selectin and VE-cadherin were measured by commercially available immunoassays according to the manufacture’s recommendations. Adrenaline (2-CAT ELISA, Labor Diagnostica Nord GmbH & Co. KG, Nordhorn, Germany; lower limit of detection (LLD) 10 pg/mL (adrenaline, normal reference < 100 pg/mL), Syndecan-1 (Diaclone Nordic Biosite, Copenhagen, Denmark; LLD 4.94 ng/mL), soluble thrombomodulin (Nordic Biosite, Copenhagen, Denmark; LLD 0.31 ng/mL), soluble E-selectin (IBL International GMBH, Hamburg, Germany; LLD 0.3 ng/mL) and soluble VE-cadherin (R&D Systems Europe, Ltd., Abingdon, UK; LLD 0.113 ng/mL).

An automated analyzer (Sysmex CA-CS100i System; Siemens AG) analyzed the following coagulation factor activities (normal range): Factor II (FII: 0.78–1.17 IU/mL), factor V (FV: 0.66–1.14 IU/mL), factor VII (FVII: 0.150–1.50 IU/mL), factor VIII (FVIII: 0.52–1.53 IU/mL), factor IX (FIX: 0.58–1.38 IU/mL), factor X (FX: 0.50–1.50 IU/mL), factor XI (FXI: 0.50–1.50 IU/mL), factor XIII (FXIII: 0.70–1.40 IU/mL), vW antigen (vWF Ag: 0.50–1.60 IU/mL), protein C (PC: 0.75–1.34 IU/mL), antithrombin (AT: 0.80–1.30 IU/mL).

Prothrombin fragment 1 + 2 (PT Frag 1 + 2; Enzygnost® F 1 + 2 (monoclonal); Siemens Healthcare Diagnostics Products GmbH, Marburg, Germany), tissue plasminogen activator (tPA; Asserachrom® tPA, Diagnostica Stago, Asnières sur Seine, France), plasminogen activator inhibitor-1 (PAI-1; Asserchrom® PAI-1; Diagnostica Stago), plasmin-α2-antiplasmin complex (PAP; PAP micro ELISA; DRG Instruments GmbH, Marburg, Germany), urokinase (uPA, Abcam, UK) (Abbexa, Cambridge, UK) were measured using sandwich enzyme-linked immunosorbent assays (ELISAs). Fibrinogen levels (Siemens Thrombin reagent, Sysmex UK) and α2-antiplasmin (Siemens Berichrom α2-antiplasmin; Sysmex UK) were determined in the hospital laboratories with a Sysmex CS2100i automated analyzer (Sysmex UK) according to standard protocols. Latex immunoassays were used to quantify the levels of D-dimer (Siemens Innovance D-dimer; Sysmex UK) also with the Sysmex CS2100i automated analyzer.

### SNP analyses

DNA was isolated from buffy-coat by QiaGen FlexiGen protocol and it was screened by means of a multiplex TaqMan-based analysis on a Via7 instrument (Applied Biosystems). Single Nuclear Polymorphism (SNP) assays of the β2-adrenergic receptor: Rs 1,042,713; Rs 1,042,714; Rs 1,042,717; Rs 1,800,888, thrombomodulin: Rs 1962; Rs 1,042,580; Rs 3,176,123, endothelial protein C receptor (EPCR): Rs 867,186, heparanase: Rs 4,364,254; Rs 4,693,608, E-selectin: Rs 1,805,193; Rs 5361 and Fibrinogen: Rs 2,020,918 that were validated on 10 samples each by direct sequencing of PCR products before analyses. The SNP´s focused on the glycocalyx, the protein C system, the sympathetic adrenergic system which are the parts of SHINE and E-selectin, an activation marker of the endothelium, together with fibrinogen, which is a pivotal part of TIC.

### SHINE phenotypes

The SHINE phenotypes were developed using the biomarkers adrenaline, syndecan-1, and sTM, reflecting the level of sympathetic activation, degree of glycocalyx damage and severity of impairment of the protein C system, as previously described [7]. The data was converted to ng/ml and normalized by log2 and further Pareto scaled before creating a heatmap with an unsupervised hierarchical clustering algorithm using a dendrogram with the Euclidian distance measure and the ward cluster algorithm.

In total, 11 patients had an adrenaline value below LLD and were considered missing values, i.e., 3.5% of the total population. To handle this, a random forest imputing approach was applied on the datafile of all measured biomarkers (adrenaline, noradrenaline, syndecan-1, sTM, sE-selectin, and VE-cadherin) using Missforest package in R [17], allowing a minimally altering of the biomarker characteristics.

### Statistical analyses

Statistical analyses were performed in RStudio 2022.07.1. Group characteristics and biomarkers were compared by Kruskal–Wallis test or Pearson Chi-Square test with unadjusted p-values reported. Post-hoc pairwise comparisons were done by Kruskal–Wallis test or Fisher’s test, adjusted for multiple testing by the Holm-Bonferroni method. Adjusted p-values < 0.05 were considered significant.

Analysis of the 20 measured pro -and anti-coagulation -and fibrinolytic factors contribution to the SHINE phenotypes was assessed by partial least squares-discriminant analysis (PLS-DA) to identify the importance of the variables among the phenotypes (VIP score). In total, 6.2% of features were missing the coagulation biomarkers data set. Missing values were imputed using the Missforest package in R and were log2, and Pareto scaled prior to the PLS-DA analysis.

## Results

### Patient characteristics

A total of 313 trauma patients were included in this study, with a median age of 46 years and 75% being males (Table 1). The vast majority of patients suffered blunt trauma, and approximately half of the patients presented with a TBI. The median ISS was 25, and 44% received a blood transfusion within the first 12 h. The 24-h and 28-day mortality was 11% and 21%, respectively.

**Table 1:** Demographics

Patient and injury characteristics	
Age, years – median (Q1; Q3)	46 (31; 59)
Male sex	75%
Penetrating trauma	7%
Traumatic brain injury	55%
AIS Head & Neck – median (Q1; Q3)	3 (0; 4)
AIS Face – median (Q1; Q3)	0 (0; 1)
AIS Thorax – median (Q1; Q3)	3 (0; 4)
AIS Abdomen – median (Q1; Q3)	0 (0; 2)
AIS Extremity – median (Q1; Q3)	2 (0; 3)
AIS External – median (Q1; Q3)	0 (0; 0)
ISS – median (Q1; Q3)	25 (20; 34)
Admission characteristics	
Heart rate, bpm – median (Q1; Q3)	90 (75; 110)
SBP, mmHg – median (Q1; Q3)	124 (96; 145)
GCS – median (Q1; Q3)	13 (6; 15)
Hemoglobin (g/L) – median (Q1; Q3)	13.4 (12.1; 14.5)
Platelet count (10^9^/L) – median (Q1; Q3)	217 (173; 260)
INR – median (Q1; Q3)	1.1 (1; 1.2)
APTT, s – median (Q1; Q3)	26 (23; 32)
Lactate, mmol/L – median (Q1; Q3)	2.2 (1.4; 3.9)
BE, mEq/L – median (Q1; Q3)	-3.4 (-7.2; -1)
Syndecan-1, ng/mL – median (Q1; Q3)	37 (21; 90)
sTM, ng/mL – median (Q1; Q3)	3.0 (1.9; 4.1)
Adrenaline, pg/mL – median (Q1; Q3)	201 (79; 356)
sE-selectin– median (IQR)	63 (46 – 89)
sVE-cadherin – median (IQR)	6.0 (3.6 – 8.5)
Transfusions at 12 h*	
Transfused (1 + units) – median (Q1; Q3)	44%
Major hemorrhage (4 + units) – median (Q1; Q3)	31%
Massive hemorrhage (20 + units) – median (Q1; Q3)	11%
Outcome	
Ventilator days at 28d – median (Q1; Q3)	1 (0; 5)
Vasopressor days at 28d – median (Q1; Q3)	0 (0; 2)
RRT days at 28d – median (Q1; Q3)	0 (0; 0)
VTE	5%
SOFA score at 24 h – median (Q1; Q3)	8 (4; 10)
ICU LOS – median (Q1; Q3)	3 (1; 8)
Hospital LOS – median (Q1; Q3)	11 (5; 24)
ICU-free days at 28d – median (Q1; Q3)	22 (4; 27)
Hospital-free days at 28d – median (Q1; Q3)	7 (0; 19)
24 h mortality	11%
28d mortality	21%

*AIS* Abbreviated Injury Scale; *APTT* Activated partial thromboplastin time; *BE* Base excess; *GCS* Glasgow coma scale; *ICU* Intensive care unit; *INR* International normalized ratio; *ISS* Injury severity score; *LOS* Length of stay; *RRT* Renal replacement therapy; *SBP* Systolic blood pressure, *SOFA* Sequential organ failure assessment; *VTE* Venous thromboembolism

*Packed red blood cells, fresh frozen plasma, cryoprecipitate, platelets

### SHINE phenotypes

**Fig. 1:**
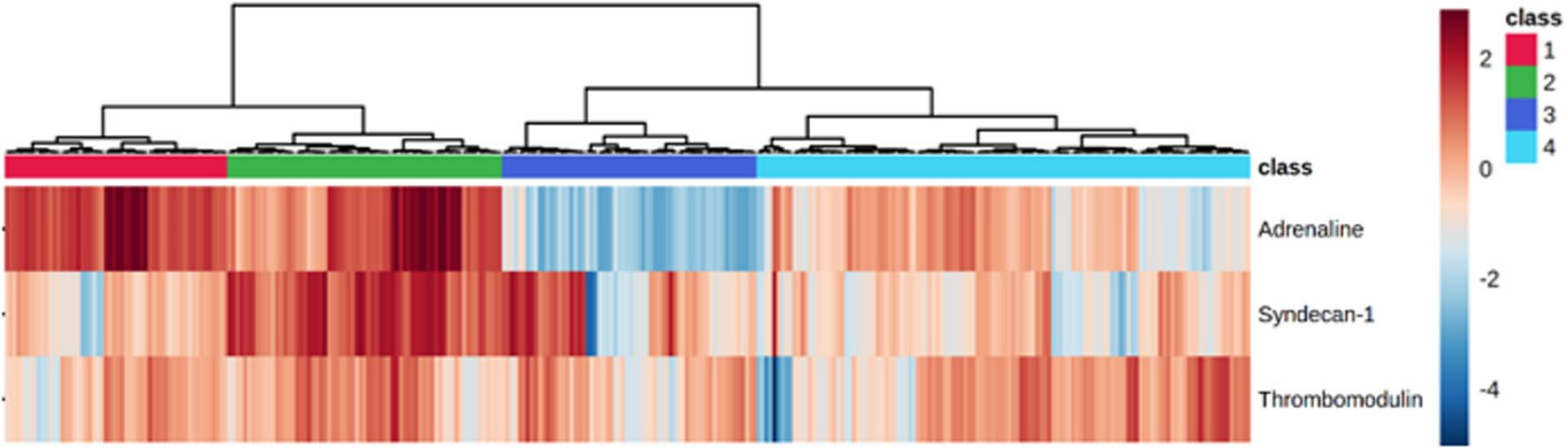
Identification of four SHINE phenotypes according to baseline plasma levels of adrenaline, syndecan-1, and soluble thrombomodulin

A minimum of four distinct SHINE phenotypes, defined by the levels of syndecan-1, sTM, and adrenaline, were identified by the hierarchical clustering algorithm (Fig. 1).

The clinical characteristics of the four SHINE phenotypes are presented in Table 2. **Phenotype 1** presented with significantly higher level of adrenaline than the other three phenotypes and 28-day mortality was 23%. **Phenotype 2** had an ISS similar to the other phenotypes. The syndecan-1 level was significantly higher in phenotype 2 than in the other phenotypes. The APTT, base deficit and incidence of massive hemorrhage was significantly higher in phenotype 2 than the other phenotypes. The 28-day mortality in phenotype 2 was 39% being significantly higher than phenotype 3 and 4 and ~ 70% higher than phenotype 1. **Phenotype 3** had significantly lower heart rate than phenotype 1 and 2 and significantly lower adrenaline levels than the other phenotypes. 28-day mortality was 15%. **Phenotype 4** had lower ISS than phenotype 2 and 28-day mortality was 14%.

**Table 2:** Demographics by endothelial phenotype

	Phenotype 1 (n = 56)	Phenotype 2 (n = 69)	Phenotype 3 (n = 64)	Phenotype 4 (n = 124)	*p*
Patient and injury characteristics					
Age, years – median (IQR)	42 (32–57)	46 (31–62)	45 (29–57)	47 (31–62)	0.73
Male sex	79%	67%	75%	77%	0.35
Penetrating trauma	11%	13%	4%	4%	0.11
Traumatic brain injury	45%	54%	67%	53%	0.09
AIS Head & Neck – median (IQR)	2 (0; 5)	3 (0; 4)	3 (0; 4)	3 (0; 4)	0.41
AIS Face – median (IQR)	0 (0; 0)^3^	0 (0; 0)^3^	0 (0; 2)^1,2^	0 (0; 1)	0.01
AIS Thorax – median (IQR)	3 (0; 4)	3 (0; 4)	2 (0; 3)	3 (0; 4)	0.26
AIS Abdomen – median (IQR)	0 (0; 2)	0 (0; 3)	0 (0; 2)	0 (0; 2)	0.09
AIS Extremity – median (IQR)	2 (0; 3)	2 (0; 3)	1 (0; 3)	2 (0; 3)	0.22
AIS External – median (IQR)	0 (0; 0)	0 (0; 0)	0 (0; 0)	0 (0; 0)	0.83
ISS – median (IQR)	25 (20–35)	29 (25–41)^4^	25 (20–34)	25 (19–30)^2^	0.02
Admission characteristics					
Heart rate, bpm – median (IQR)	95 (80–117)^3^	100 (81–120)^3,4^	80 (70–102)^1,2^	88 (72–100)^2^	< 0.001
SBP, mmHg – median (IQR)	117 (85–140)	120 (92–143)	125 (99–141)	130 (106–149)	0.04
GCS – median (IQR)	13 (8–15)	12 (6–15)	12 (4–15)	13 (6–15)	0.68
Hemoglobin (g/L) – median (IQR)	13.7 (12.5–14.3)	12.9 (10.4–14.3)	13.2 (11.8–14.5)	13.7 (12.6–14.5)	0.05
Platelet count (10^9/L) – median (IQR)	236 (192–259)	205 (159–251)	226 (181–265)	217 (169–262)	0.24
INR – median (IQR)	1.1 (1.0–1.1)	1.1 (1.0–1.3)	1.1 (1.0–1.2)	1.1 (1.0–1.1)	0.05
APTT, s – median (IQR)	26 (23–30)^2^	34 (26–46)^1,3,4^	26 (23–29)^2^	25 (23–30)^2^	< 0.001
Lactate, mmol/L – median (IQR)	2.7 (1.5–4.6)	3.3 (2.0–6.7)^3,4^	1.9 (1.3–2.6)^2^	2.1 (1.3–3.2)^2^	< 0.001
Base deficiency, mEq/L – median (IQR)	3.7 (2.1–7.6)^2^	6.6 (3.5–11)^1,3,4^	2.3 (1.0–4.3)^2^	2.7 (0.6–5.4)^2^	< 0.001
sE-selectin– median (IQR)	56 (44–81)	65 (51–84)	60 (42–95)	67 (47–95)	0.34
sVE-cadherin– median (IQR)	5.8 (3.1–8.2)	5.0 (2.9–7.4)^3,4^	6.3 (4.9–8.8)^2^	6.2 (4.0–9.4)^2^	0.06
SHINE biomarkers					
Adrenaline, pg/mL – median (IQR)	1370 (1080–1700)^2,3,4^	950 (409–2260)^1,3,4^	19 (13–27)^1,2,4^	201 (79–356)^1,2,3^	< 0.001
Syndecan-1, ng/mL – median (IQR)	34 (22–47)^2^	150 (93–216)^1,3,4^	41 (18–115)^2,4^	25 (16–38)^2,3^	< 0.001
sTM, ng/mL – median (IQR)	2.7 (1.9–3.3)	3.5 (2.3–4.5)	2.7 (1.8–3.6)	3.1 (1.9–4.3)	0.06
Transfusions at 12 h*					
Transfused (1 + units) – median (IQR)	55%^4^	68%^3,4^	36%^2^	31%^1,2^	< 0.001
Major hemorrhage (4 + units) – median (IQR)	38%	53%^3,4^	22%^2^	21%^2^	< 0.001
Massive hemorrhage (20 + units) – median (IQR)	7%^2^	27%^1,3,4^	6%^2^	7%^2^	< 0.001
Outcome					
Ventilator days at 28d – median (IQR)	1 (0–5)	1 (0–5)	1 (0–7)	1 (0–5)	0.77
Vasopressor days at 28d – median (IQR)	0 (0–4)	0 (0–1)	0 (0–2)	0 (0–2.5)	0.43
RRT days at 28d – median (IQR)	0 (0–0)	0 (0–0)	0 (0–0)	0 (0–0)	0.40
SOFA score at 24 h – median (IQR)	9 (6–11)	8 (5–10)	6 (4–9)	8 (5–10)	0.19
ICU LOS, days – median (IQR)	2 (0–8)	3 (0–7)	4 (1–9)	2 (1–8)	0.32
Hospital LOS, days – median (IQR)	14 (7–29)	8 (2–20)	13 (6–25)	11 (5–24)	0.12
ICU-free days at 28d – median (IQR)	21 (8–27)	12 (0–25)^4^	23 (12–26)	24 (10–27)^2^	0.01
Hospital-free days at 28d – median (IQR)	2 (0–16)	0 (0–14)^4^	9 (0–20)	10 (0–20)^2^	0.02
Venous thromboembolism	0%	4%	5%	7%	0.20
24 h mortality	14%	23%^3,4^	3%^2^	6%^2^	< 0.001
28d mortality	23%	39%^3,4^	14%^2^	15%^2^	< 0.001

*AIS* Abbreviated Injury Scale; *APTT* Activated partial thromboplastin time; *BE* Base excess; *GCS* Glasgow coma scale; *ICU* Intensive care unit; *INR* International normalized ratio; *ISS* Injury severity score; *LOS* Length of stay; *RRT* Renal replacement therapy; *SBP* Systolic blood pressure, *SOFA* Sequential organ failure assessment

*Packed red blood cells, fresh frozen plasma, cryoprecipitate, platelets

^1,2,3,4^Superscripted numbers indicate statistically significant differences in post-hoc pairwise comparison of individual endothelial phenotypes (i.e., *p* < 0.05 adjusted for multiple testing)

### Markers of coagulation -and fibrinolysis in the SHINE phenotypes

Phenotype 2 had significantly lower prothrombin, factor V, IX, X, XI, free protein S Ag and antithrombin levels as well as increased EXTEM CT compared to the other phenotypes (Table 3). Phenotype 2 also had significantly increased fibrinolysis evident by higher PAP, tPA and lower alpha 2-antiplasmin than the other phenotypes. In the remaining phenotypes, apart from PAP, which was significantly lower in phenotype 3- and 4 compared to phenotype 1, no significant differences in any of the coagulation- and fibrinolytic markers were observed.

**Table 3:** Coagulation biomarkers by endothelial phenotype

	Phenotype 1 (n = 56)	Phenotype 2 (n = 69)	Phenotype 3 (n = 64)	Phenotype 4 (n = 124)	*p*
Baseline coagulation tests					
Fibrinogen – median (IQR)	1.6 (1.3–2.4)	1.4 (0.9–1.9)^3,4^	1.8 (1.3–2.3)^2^	1.9 (1.4–2.5)^2^	0.002
Prothrombin – median (IQR)	80 (66–95)^2^	70 (51–83)^1,3,4^	79 (68–90)^2^	83 (69–97)^2^	< 0.001
Factor V – median (IQR)	58 (37–77)^2^	40 (18–62)^1,3,4^	60 (40–78)^2^	70 (53–87)^2^	< 0.001
Factor VII – median (IQR)	86 (76–99)^2^	75 (58–96)^1,4^	82 (63–104)	82 (69–104)^2^	0.02
Factor VIII – median (IQR)	274 (152–354)	184 (107–293)	218 (137–319)	209 (137–305)	0.10
Factor IX – median (IQR)	96 (76–121)^2^	80 (55–100)^1,3,4^	100 (83–126)^2^	99 (80–116)^2^	< 0.001
Factor X – median (IQR)	82 (66–97)^2^	67 (44–82)^1,3,4^	78 (64–95)^2^	81 (67–97)^2^	< 0.001
Factor XI – median (IQR)	94 (70–127)^2^	68 (44–95)^1,3,4^	86 (71–112)^2^	90 (74–110)^2^	< 0.001
Factor XIII – median (IQR)	94 (80–114)	93 (69–115)	103 (86–124)	103 (85–120)	0.11
vWF Ag – median (IQR)	226 (179–314)	247 (200–332)	221 (163–282)	225 (162–282)	0.08
PF 1 + 2, pmol/L – median (IQR)	2520 (1560–4390)	2500 (1140–5530)	2160 (1060–3260)	1740 (1000–3290)	0.06
Protein C – median (IQR)	84 (73–101)	78 (62–100)	85 (73–104)	88 (71–108)	0.13
Free Protein S Ag – median (IQR)	87 (69–108)^2^	71 (50–101)^1,3,4^	92 (76–112)^2^	92 (71–114)^2^	0.003
Antithrombin – median (IQR)	84 (68–98)^2^	66 (48–90)^1,3,4^	82 (70–94)^2^	81 (64–92)^2^	0.001
tPA, ng/mL – median (IQR)	14 (9.2–25)^2^	21 (17–33)^1,3,4^	17 (11–24)^2^	15 (9.5–20)^2^	< 0.001
PAP, 1000 ug/L – median (IQR)	7.2 (4.5–13)^2,3,4^	13 (5.8–20)^1,3,4^	4.6 (2.4–9.7)^1,2^	4.9 (2.5–9.6)^1,2^	< 0.001
Alpha 2-antiplasmin – median (IQR)	74 (53–91)^2^	49 (28–74)^1,3,4^	80 (63–100)^2^	81 (57–98)^2^	< 0.001
PAI-1, ng/mL – median (IQR)	23 (16–33)	24 (17–36)	27 (18–42)	22 (14–35)	0.30
D-dimer, 10^3 ng/ml – median (IQR)	32 (19–69)	27 (8.0–96)	22 (8.3–52)	18 (6.5–55)	0.15
Fibrin monomers – median (IQR)	199 (116–295)	186 (87–252)	200 (95–308)	148 (51–228)	0.05
Baseline viscoelastic tests					
EXTEM CT, s – median (IQR)	59 (49–70)^2^	69 (56–88)^1,3,4^	59 (53–64)^2^	59 (52–67)^2^	0.01
EXTEM Alpha angle – median (IQR)	72 (68–74)	70 (65–74)	71 (67–75)	72 (68–74)	0.12
EXTEM CFT, s – median (IQR)	91 (78–111)	108 (85–135)^4^	95 (76–115)	92 (80–112)^2^	0.03
EXTEM MCF, mm – median (IQR)	62 (61–67)	60 (54–64)^3,4^	62 (59–68)^2^	63 (58–67)^2^	0.01
EXTEM time to MCF, s – median (IQR)	1750 (1550–1870)	1750 (1540–2020)	1730 (1550–1930)	1700 (1520–1910)	0.83
EXTEM Li30, % – median (IQR)	100 (100–100)	100 (100–100)	100 (100–100)	100 (100–100)	0.76
FIBTEM MCF, mm – median (IQR)	13 (10–18)	12 (8–15)	13 (10–17)	13 (11–17)	0.05

*CFT* Clot formation time; *CT* Clot time; *Li30* Lysis index at 30 min; *MCF* Maximum clot firmness, *PAI-1* Plasminogen activator inhibitor-1; *PAP* Plasmin-alpha-2-antiplasmin complex; *PF* Prothrombin fragment; *TEM* Thromboelastometry, *tPA* Tissue plasminogen activator; *vWF* Von Willebrand Factor

^1,2,3,4^Superscripted numbers indicate statistically significant differences in post-hoc pairwise comparison of individual endothelial phenotypes (i.e., *p* < 0.05 adjusted for multiple testing)

### Discriminators of the SHINE phenotypes

**Fig. 2:**
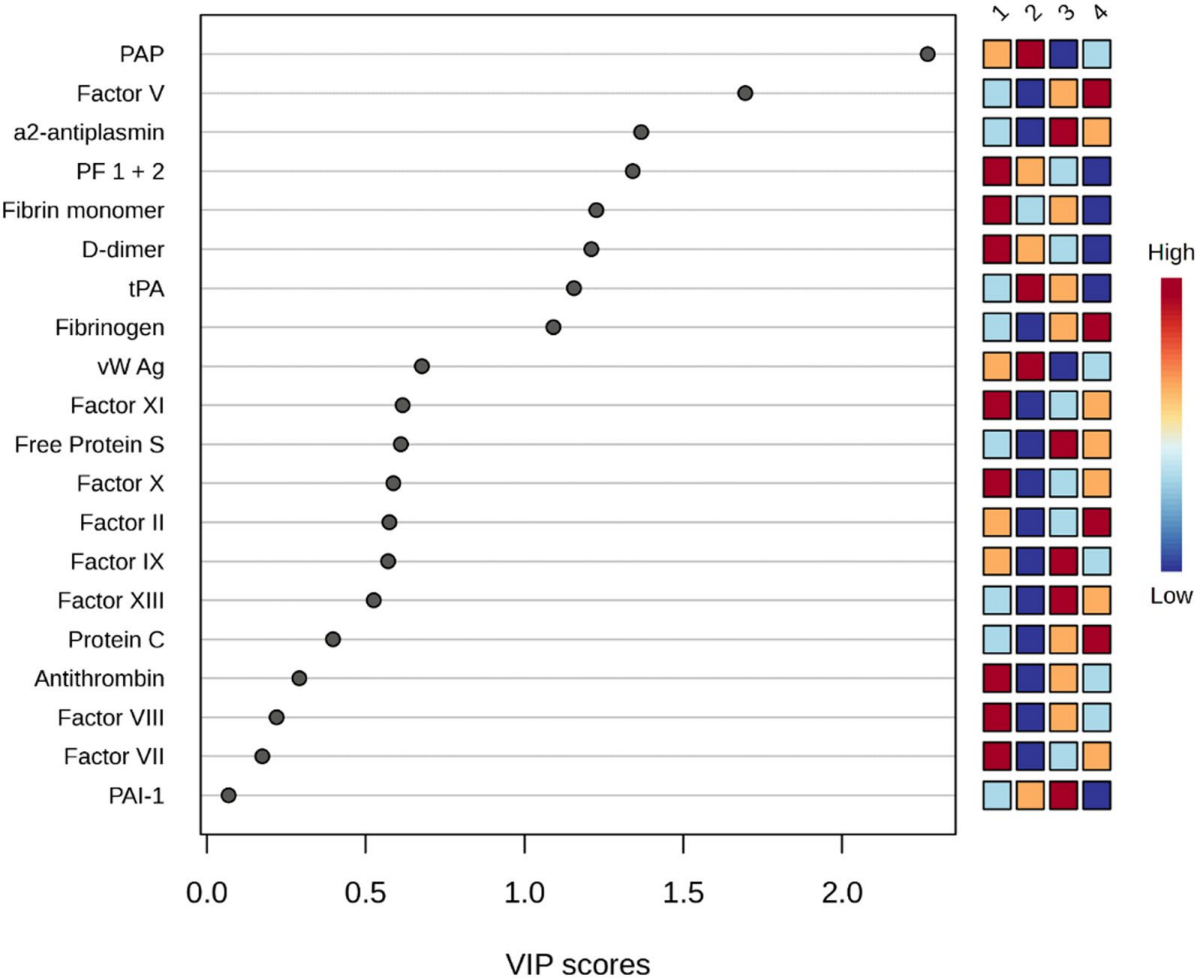
PLS-DA analysis of coagulation -and fibrinolytic factors in the different SHINE phenotypes

The PLS-DA analysis showed that the top discriminator between the SHINE phenotypes was the plasmin – alpha 2-antiplasmin (PAP) complex followed by Factor V with VIP scores above 2 and 1.5, respectively (Fig. 2). Both were also significantly different between phenotype 2 and the other phenotypes (Table 3). Alpha 2-antiplasmin was the third highest discriminator between the SHINE phenotypes and was also significantly different between phenotype 2 and the other phenotypes (Table 3).

### Singe nucleotide variations in the SHINE phenotypes

**Fig. 3:**
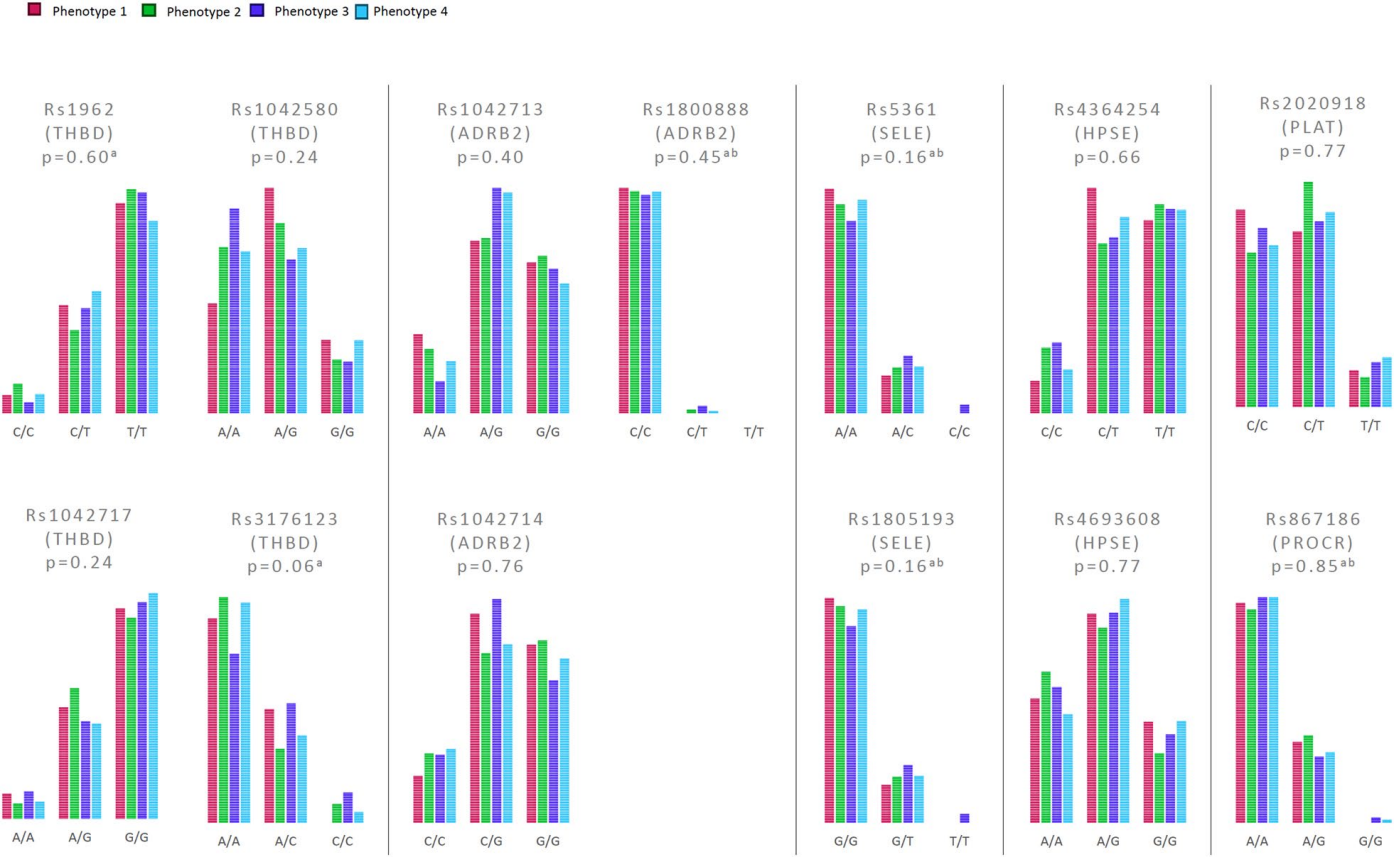
SNP analyses of the β2-adrenergic receptor, thrombomodulin, endothelial protein C receptor (EPCR), heparanase, E-selectin and Fibrinogen

We found no significant differences between SHINE phenotypes concerning the SNP’s investigated, Fig. 3.

## Discussion

Hierarchical clustering of the SHINE biomarkers adrenaline, syndecan-1 and sTM identified four phenotypes of which SHINE phenotype 2, encompassing 22% of the trauma patients, was characterized by significantly higher glycocalyx shedding and significantly more hypocoagulable- and hyperfibrinolytic than the other three phenotypes. The mortality in phenotype 2 was significantly higher than phenotypes 3- and 4 and 70% higher than phenotype 1, whereas the ISS in phenotype 2 was similar to phenotypes 1- and 3. Interestingly, no significant difference in coagulation- and fibrinolytic markers were observed between phenotypes 1, 3, -and 4, confining the coagulopathy of trauma to phenotype 2. Regarding sTM only a trend towards a difference between the phenotypes was observed and, hence, it is unlikely that the observed differences in coagulopathy and mortality between phenotype 2 and the other phenotypes is caused by variations in the activity in the protein C system.

SHINE builds on the premise that increasing injury severity leads to increased sympathetic activation with release of progressively higher concentrations of catecholamines leading to more severe endothelial damage and higher mortality [7, 18]. In this study we found a non-linear relationship between sympathetic activation and endotheliopathy in SHINE, best illustrated by that trauma patients belonging to different phenotypes presented with the highest levels of adrenaline (phenotype 1) and highest level of glycocalyx shedding (phenotype 2), respectively. Similarly, phenotype 3 displayed an almost blunted sympathetic response with significantly lower adrenaline levels than the other phenotypes but similar level of glycocalyx shedding as phenotype 1 and higher than phenotype 4.

The shock severity, alone, cannot explain the high glycocalyx shedding in phenotype 2 as only base deficiency was significantly higher, whereas systolic blood pressure and lactate was not significantly different compared to the other SHINE phenotypes. Similarly, the ISS was not significantly higher in phenotype 2 that phenotype 1 – and 3 and, therefore, not alone can explain the high glycocalyx shedding. The finding that phenotype 2, in addition to higher glycocalyx shedding, also presented with the highest mortality is in alignment with previous reports [10, 11, 13] A novel finding, though, was that it is confined to approximately 20% of the whole trauma population. Collectively, this suggests that other factors than sympathetic activation, ISS and shock severity are involved in the pathophysiology of high glycocalyx shedding. We did, however, not find any significant differences in the SNP´s investigated between the phenotypes. As only a few SNP´s were investigated this does not exclude the possibility of a genetic component contributing to both the sympathetic response and the level of glycocalyx shedding in the different phenotypes.

The most striking difference between the four SHINE phenotypes was the severity of coagulopathy observed in phenotype 2 being significantly more hypocoagulable -and hyperfibrinolytic than the other phenotypes. Phenotype 2 had significantly lower levels of coagulation factors involved primarily in the generation of the thrombin burst i.e., factor II, V, IX, X and XI, which is responsible for the conversion of fibrinogen into fibrin being a pivotal part of the clot. It has previously been reported that trauma patients with high glycocalyx shedding are hypocoagulable as measured by APTT and increased reaction/clotting time as measured by whole blood viscoelastic assays translating into higher transfusion requirements [9–11, 13], similar to phenotype 2. Endogenous heparinization has been proposed as a potential mechanism for this hypocoagulable state secondary to the release of large amount of heparan sulphate by shedding of the glycocalyx but this warrants further investigation [12].

Hyperfibrinolysis has consistently been associated to the most severely injured trauma patients and this is associated with massive hemorrhage and high mortality [8, 11, 19, 20] similar to phenotype 2 except that the injury severity was not significantly different from phenotype 1 and 3. A potential explanation for these different observations may be that only patients with ISS above 15 was included in the present study and that the patients were not stratified based on various assessments of the level of fibrinolysis [21, 22]. Despite that phenotype 2 had severe hyperfibrinolysis evident by high tPA and PAP levels and low alpha 2-antiplasmin level as well as clinically by increased transfusion requirements no difference with regard to ROTEM EXTEM Li30 was observed when compared to the other phenotypes and this is in alignment with previous reports [22–24]. This finding questions the utility of EXTEM Li30 to identify clinically important hyperfibrinolysis in trauma patients and, in particular in identifying SHINE phenotype 2, warranting further investigation.

PLS-DA analysis identified PAP, FV and alpha2-antiplasmin as the top three features discriminating between the phenotypes. FV is activated by thrombin into FVa which is a cofactor of the prothrombinase complex consisting also of FXa which convert prothrombin to thrombin on cell surface membranes [25]. The prothrombinase complex can catalyze the activation of prothrombin at a rate 3 × 10^5^-fold faster than can Factor Xa alone [26]. Thus, FVa is required for an efficient thrombin burst and the reduced levels observed in phenotype 2 may be the main cause of hypocoagulability. Furthermore, if this is confirmed it could be speculated whether these patients would benefit from higher concentrations of FV than what is readily available in standard plasma units, being the only available therapeutic option currently, but this requires further investigation [27].

The high levels of PAP and tPA and low levels of alpha2-antiplasmin reflects significantly increased plasmin levels in phenotype 2 and plasmin degrades the fibrin clot, effectively preventing hemostasis.

The present study has important limitations. It is a retrospective observational study and, therefore, no causality concerning any of the findings can be inferred. Furthermore, it encompasses a limited number of patients admitted to European trauma centers at tertiary university hospitals only. Only patients with ISS above 15 were included in this study so our findings cannot be generalized to the whole spectrum of trauma patients. Also, the patients were included a decade ago which may influence the results presented. Although the hemostatic system was extensively investigated here it cannot be excluded that other factors involved in hemostasis would be relevant to include in this study and similarly only a limited number of SNP´s were investigated and important differences in genetic variations between the SHINE phenotypes not identified here cannot be excluded. The potential contribution of the platelets to the identified factor V induced hypocoagulability cannot be fully elucidated although no significant differences between phenotypes with regard to platelet count or maximum clot strength assessed by whole blood viscoelastic ROTEM® was observed. Further limitations include that the study population had a low degree of severe abdominal injury (based on AIS) compared with other critically ill trauma cohorts and single vital sign measurements were used to characterize patient's shock physiology, which may result in misclassification bias. Also, the lack of information on blood transfusion and other resuscitative treatments, which may modify the association between SHINE/coagulation phenotypes and outcome. Also, not having data on the incidence of pre-hospital tranexamic acid or blood product administration in the four SHINE phenotypes is a further limitation.

In conclusion, hierarchical clustering analysis of syndecan-1, sTM and adrenaline identified four distinct SHINE phenotypes. The trauma induced coagulopathy with profound hypocoagulability and hyperfibrinolysis, increased transfusion requirements and high mortality was confined to only one SHINE phenotype, encompassing only 20% of the whole trauma cohort warranting further investigation.

## Data Availability

Anonymised data are available upon request to the corresponding author.

## Abbreviations

SHINE: Shock-Induced Endotheliopathy
PAP: Plasmin-alpha-antiplasmin
ISS: Injury severity score
TBI: Traumatic brain injury
aPTT: Activated partial thromboplastin time
PT: Prothrombin time
ROTEM: Rotational thromboelastometry
sTM: Soluble thrombomodulin
EC: Endothelial cells
ACIT-2: A retrospective multicenter observational cohort study, the Activation of Coagulation and Inflammation in Trauma-2
SNP: Single nucleotide polymorphisms
TIC: Trauma induced coagulopathy
EPCR: Endothelial protein C receptor
LLD: Lower limit of detection
tPA: Tissue plasminogen activator
PAI-1: Plasminogen activator inhibitor-1
ELISAs: Enzyme-linked immunosorbent assays
PLS-DA: Partial least squares-discriminant analysis
AIS: Abdominal injury severity score
